# Cyclosporine A trough concentrations are associated with acute GvHD after non-myeloablative allogeneic hematopoietic cell transplantation

**DOI:** 10.1371/journal.pone.0213913

**Published:** 2019-03-21

**Authors:** Elizabeth A. de Kort, Heleen S. de Lil, Manita E. J. Bremmers, Lenneke F. J. van Groningen, Nicole M. A. Blijlevens, Gerwin Huls, Roger J. M. Brüggemann, Suzanne van Dorp, Walter J. F. M. van der Velden

**Affiliations:** 1 Department of Hematology, Radboud University Medical Center, Nijmegen, The Netherlands; 2 Department of Hematology, University Medical Center Groningen, Groningen, The Netherlands; 3 Department of Pharmacy, Radboud University Medical Center, Nijmegen, The Netherlands; Istituto Di Ricerche Farmacologiche Mario Negri, ITALY

## Abstract

Low plasma CsA concentrations (<300–350 ng/mL) early following allogeneic hematopoietic stem cell transplantation (HSCT) is associated with an increased risk of developing acute graft-versus-host disease (aGvHD). Nevertheless, the current optimal target trough concentration for CsA following HSCT is considered to be 200–400 ng/mL. Here, we performed a retrospective analysis of a homogeneous group of 129 patients who received HSCT after non-myeloablative conditioning, and we analyzed the impact of CsA trough concentration measured during the first four weeks (CsA W1-4) on the incidence aGvHD, relapse-free survival (RFS), non-relapse mortality (NRM), overall survival (OS), and toxicity. The 180-day incidence of grade II-IV aGvHD was 25% (32/129 patients). In multivariate analysis the incidence of grade II-IV aGvHD was significantly lower among patients with a CsA W1-4 concentration ≥350 ng/mL compared to patients with a concentration <350 ng/mL (18% versus 38%, respectively; *P* = 0.007), with a hazard ration (HR) of 0.38 (95% CI: 0.19–0.77). In contrast, we found no correlation between CsA trough concentration and RFS, NRM, or OS. Moreover, we found an increased incidence of hypomagnesemia at higher CsA concentrations, but no difference in the incidence of acute renal toxicity, hepatic toxicity, or electrolyte imbalance. Interestingly, 30% of patients experienced hyponatremia with no apparent cause other than the use of CsA, with urinalysis suggesting SIADH as the underlying cause. Our findings suggest that a CsA trough concentration of 350–500 ng/mL might be more appropriate in the first month following non-myeloablative HSCT.

## Introduction

Although allogeneic hematopoietic stem cell transplantation (HSCT) is a potentially curative treatment for many hematological malignancies, it is generally associated with significant early post-transplant complications, including acute graft-versus-host disease (aGvHD), which contribute to non-relapse mortality (NRM)[1–4]. A variety of patient- and treatment-related factors can affect the incidence of these complications and include patient age, comorbidity, conditioning intensity, and the use of T cell depletion therapy. In addition, post-HSCT patients receive multiple drugs that cause side effects. Notably, the calcineurin inhibitors cyclosporine A (CsA) and tacrolimus (TAC), which are commonly used to prevent GvHD, can cause acute renal failure, hyperbilirubinemia, and electrolyte imbalances.

Calcineurin inhibitor‒based regimens are widely used and are currently considered the backbone of GvHD prophylaxis[5]. Despite the common use of these agents, the incidence of grade II-IV aGvHD among patients is approximately 40–60%. Several dosing schemes have been developed in an attempt to customize the intensity and duration of post-transplant immunosuppression in order to match the anticipated risks of GvHD and disease relapse. Historically, the ideal target trough concentration of CsA in the plasma following HSCT was 200–400 ng/mL [6–8], however, in a more recent EBMT-ELN guideline, achieving a trough concentration between 200–300 ng/mL was advised in order to prevent aGvHD early after HSCT[9]. As for side effects, a concentration up to 500 ng/mL is presumed to be safe, despite a lack of clear evidence to support this assumption[10, 11]. Low plasma concentrations of CsA and TAC—particularly in the first weeks following HSCT—have consistently been associated with a higher risk of developing GvHD[12–16]; therefore, trough concentration‒based dosing has become relatively common following HSCT. Importantly, however, in most of the above-mentioned studies, the risk of aGvHD was consistently higher in patients with a CsA concentration well below 300–350 ng/mL, suggesting that the lower limit of 200 ng/mL may not be optimal. In addition, limited data is available regarding the correlation between CsA concentration and HSCT-associated toxicity and outcomes other than aGvHD[12, 16, 17].

Here, we examined the correlation between the CsA concentration in the first month following HSCT and the incidence of early complications and outcome in a homogenous cohort of patients who received HSCT following non-myeloablative conditioning (NMA). Specifically, our goal was to determine whether current target concentrations are optimal in clinical practice, and whether adjusting these targets is warranted in order to improve outcome and reduce the risk of aGvHD in this patient population.

### Patients and methods

#### Patients

We performed a single-center retrospective analysis of 129 out of 140 consecutive patients who received an allogeneic HSCT from January 2013 through August 2017 following NMA conditioning for a hematological malignancy. At the time of treatment, all patients provided informed consent for the prospective collection of data and samples for investigational use. This retrospective study was approved by the Institutional Review Board at Radboud University Nijmegen Medical Center.

The characteristics and features of the patients, donors, and HSCT procedures are summarized in Table 1.

**Table 1:** Patient, donor, and HCT procedure characteristics (N = 129 patients).

Characteristics	
Patient age in years, median (range)	61 (20–75)
Age ≥55 years, N (%)	100 (78)
Male/Female	70/59
Diagnosis, N (%)	
• AML	76 (59)
• High-risk MDS/CMML	25 (19)
• MM/PCL	13 (10)
• Other: NHL, CLL, aCML, ALL	15 (12)
Disease risk index, N (%)	
• Low/Intermediate	40 (31)
• High/Very high	89 (69)
HCT-CI score, median, range	3 (0–8)
HCT-CI ≥ 3	77 (60)
EBMT score, median, range	3 (1–6)
Conditioning regimen, N (%)	
• Flu-TBI	65 (50)
• Decitabine-Flu-TBI	43 (33)
• ATG-Flu-TBI	14 (11)
• ATG-decitabine-Flu-TBI	7 (6)
Donor, N (%)	
• MRD	35 (27)
• MUD	73 (56)
• MMUD	21 (17)
Stem cell source, peripheral blood, N (%)	123 (95)
CMV status patient, N (%)	
• Negative	43 (34%)
• Positive	86 (66%)
CsA W1-4, median (range)	(127.5–816.7)
CsA W1-4, mean value ≥350 ng/mL, N(%)	87 (67)

Abbreviations: AML, acute myeloid leukemia; MDS, myelodysplastic syndrome; CMML, chronic myelomonocytic leukemia; MM, multiple myeloma; PCL, plasma cell leukemia; NHL, non-Hodgkin lymphoma; CLL, chronic lymphocytic leukemia; aCML, atypical chronic myeloid leukemia; ALL, acyte lymphoblastic leukemia

#### Conditioning and post-HSCT immunosuppression

All patients received Flu-TBI conditioning consisting of 30 mg/m^2^ fludarabine on days -4, -3, and -2, and low-dose TBI (2 Gy) on day -1 (days are expressed relative to transplantation)[10, 18–20]. In the case of a mismatched unrelated donor (MMUD), the patient also underwent *in vivo* T cell depletion with 2 mg/kg/day Thymoglobulin (rabbit-ATG; Genzyme) on days -8, -7, -6, and -5. In accordance with study protocols (NCT02252107), patients with an adverse risk of developing myeloid malignancies also received 10 days of decitabine (Figure A in S1 File)[21]. On day 0, all patients received a T cell‒repleted graft with mobilized peripheral blood stem cells or bone marrow stem cells.

Post-HSCT immunosuppression included mycophenolate mofetil for 28 or 96 days for matched related donors (MRDs) and unrelated donors (both matched and mismatched), respectively, and oral CsA for 180 days. CsA was initially dosed at 4.5 mg/kg twice daily, and the dose could be adjusted in the event of clinical toxicity or in the event that a suboptimal and/or toxic CsA concentration was achieved. In the case of clinical toxicity, standard practice employed at our institution is to omit one dose of CsA and reduce subsequent doses by 12.5–25%.

#### Supportive care measures

Antimicrobial prophylaxis was according to current practice (See S1 File). Ursodeoxycholic acid was not routinely used in our practice.

#### Definitions

As part of routine care at our medical center, CsA concentration was measured 4–5 days after the starting dose, at 12 hrs after the last administration. CsA concentration was subsequently measured at least once weekly during the first 2–3 months using validated ultra-performance liquid chromatography tandem mass spectrometry (UPLC-MS/MS; Waters Corporation, Milford, MA). The optimal target trough concentration of CsA was defined as 200–400 ng/mL in accordance with general recommendations[6–8]. Accordingly, CsA concentration was considered suboptimal at <200 ng/mL, high at ≥400 ng/mL, and potentially toxic at ≥500 ng/mL[10, 11]. Changes to the dosing regimen were made at the discretion of the treating HSCT physician. CsA trough concentrations measured in weeks 1 through 4 were collected and used to calculate an average value for the entire 4-week period referred to hereafter as “CsA W1-4”. For our analysis, cut-off values were used at increments of 50 ng/mL based on data published by Rogosheske *et al*.[15]

The disease risk index (DRI), HSCT comorbidity index (HSCT-CI), and European Group for Blood and Marrow Transplantation (EBMT) risk score were determined in accordance with published scoring systems[22–24].

Outpatient clinical and laboratory assessments were performed twice weekly during the first month following HSCT, and subsequently once a week. Non-hematological adverse events, including hyperbilirubinemia, electrolyte imbalances, and/or renal failure, were graded in accordance with version 4 of the Common Terminology Criteria for Adverse Events (CTCAE). Adverse events graded as ≥2 were considered to be clinically relevant.

Post-HSCT aGvHD was defined in accordance with the criteria proposed by Harris *et al*.[25]. Clinically suspected cases of aGvHD were confirmed histologically. Classic aGVHD occurs within and late onset aGvHD beyond the first 100 days post HSCT[26]. Late onset aGvHD is a frequent occurrence with the Flu-TBI scheme due to relatively late initiation of calcineurin inhibitors tapering (around day 100), therefore we evaluated aGvHD grade II-IV at day 180 [10, 27]. Chronic GvHD (cGvHD) was scored and graded according to NIH guidelines[28]. Relapse of the hematological malignancy was classified in accordance with consensus definitions and consisted almost entirely of hematological relapses (e.g., morphological relapse)[29]. Early relapse was defined as relapse within 6 months of HSCT. The occurrence of aGvHD, non-relapse mortality (NRM), relapse-free survival (RFS), and overall survival (OS) were defined in accordance with EBMT statistical guidelines[30]. GvHD/relapse-free survival (GRFS) was defined as survival without relapse, aGvHD grade III-IV or moderate-severe cGvHD[31].

#### Statistical analysis

We used descriptive statistics to analyze the patient, donor, and HSCT characteristics. To investigate possible risk factors associated with the occurrence of grade II-IV aGvHD by day 180, relapse by day 180, moderate-severe cGvHD, and toxicity (e.g., renal toxicity, hepatic toxicity, or an electrolyte imbalance), we first performed univariate analyses; we then included any factors with a *P*-value ≤0.15 in a multivariate analysis using logistic regression. For details on the factors included in the analysis see Tables A and B in S1 File. Since no significant differences were found between the MUD and MMUD subgroups for all outcome measures we chose to merge this group in the analysis.

To examine the relationship between renal toxicity and CsA W1-4, we performed a Pearson’s correlation analysis between CsA W1-4 and the change in creatinine levels relative to baseline (prior to conditioning).

We estimated the occurrence of aGvHD, NRM, RFS, GRFS and OS using the Kaplan-Meier method.

Putative factors affecting the occurrence of GvHD, NRM, GRFS, RFS, and OS were selected for univariate analysis, and any factors with a *P*-value ≤0.15 were then incorporated in a multivariate analysis using Cox regression. For details on the factors included in the analysis see Tables A and B in S1 File.

Differences with a *P*-value of <0.05 were considered statistically significant. SPSS Statistics version 22.0 (IBM Corp., Armonk, NY) was used for statistical analyses.

## Results

A total of 129 out of 140 consecutive patients were included in our final analysis. The minimum follow-up period was 6 months, and data regarding the trough CsA concentration were available for at least three separate weeks during the first month following HSCT. From the original 140 consecutive patients, 11 patients were excluded due to the following reasons: administration of intravenous CsA (4 patients), an early switch from CsA to TAC (4 patients; 2 switched due to posterior reversible encephalopathy syndrome), and insufficient data regarding CsA concentration (3 patients).

### CsA trough concentration

**Fig 1:**
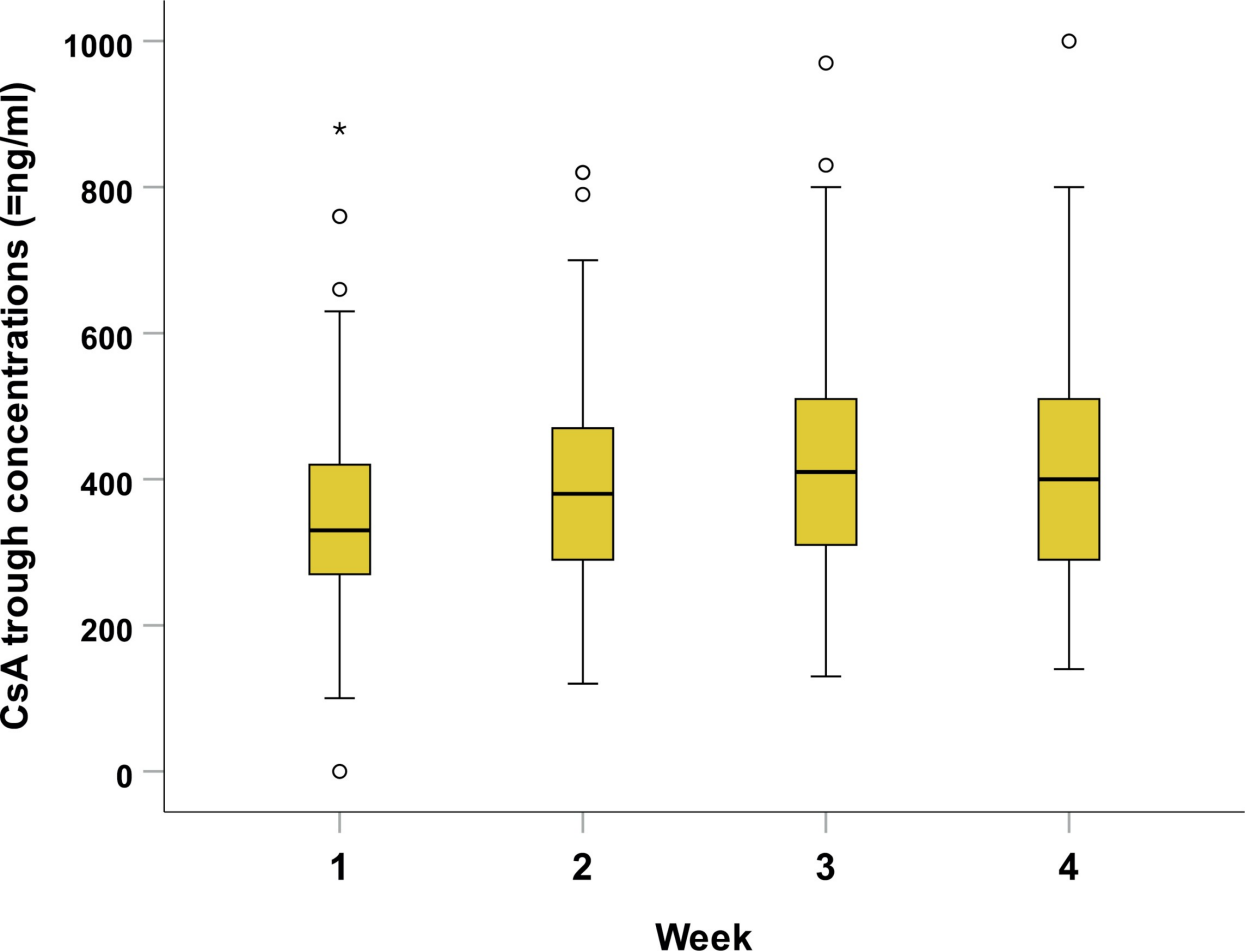
Summary of CsA trough concentration measured 1 to 4 weeks after HCT.

The median CsA trough concentration at 1, 2, 3, and 4 weeks following HSCT was 330, 380, 410, and 400 ng/mL, respectively (Fig 1). The median calculated concentration for the entire 4-week period (CsA W1-4) was 402 ng/mL (range: 127–816 ng/mL) and correlated strongly with the CsA concentration during the first 2 weeks (CsA W1-2). Based on the predefined target range of 200–400 ng/mL, 4, 65, and 24 patients had a suboptimal concentrations (i.e., <200 ng/mL), a high concentration (i.e., ≥400 ng/mL), or a potentially toxic concentration (i.e., ≥500 ng/mL), respectively.

### Post-HSCT complications

#### Acute and chronic GvHD

In our patient cohort, the median interval between HSCT and the onset of aGvHD (grade I through grade IV) was 60 days (range: 10–322 days). The 180-day rate of grade II-IV and grade III-IV aGvHD was 25% (32/129 patients) and 10% (13/129 patients), respectively (Table 2). Based on a univariate analysis, the risk factors for developing grade II-IV aGvHD were receipt of a graft from either a MUD or MMUD and a CsA W1-4 value <350 ng/mL (Table A in S1 File). A multivariate logistic regression analysis revealed that both factors had a statistically significant impact with respect to increasing the risk of grade II-IV aGvHD by day 180.

**Table 2:** Post-HCT complications and outcome (N = 129).

Complication	
Acute GvHD, N (%)	
• Grade 0-I	91 (70)
• Grade II	22 (17)
• Grade III-IV	16 (13)
Acute GvHD grade II-IV day 180, N (%)	32 (25)
Acute GvHD grade III-IV day 180, N (%)	13 (10)
Primary graft failure, no (%)	5 (4%)
CMV infection or disease <3 months, N (%)	30/2 (23/1.5)
EBV infection/disease, N (%)	6/2 (4.5/1.5)
Early hyperbilirubinemia, grade 3, N (%)	38 (29)
Onset hyperbilirubinemia, median day (range)*	6 (1–11)
Acute kidney injury, grade 2, N (%)	63 (49)
Acute kidney injury, grade 2, N (%)	10 (8)
Creatinine change from baseline, median (range)	x 2.0 (1.0–5.4)
Creatinine >25% BL, median day of onset (range)*	20 (6–42)
Electrolyte imbalance	
• Hyperkalemia grade 2, N (%)	8 (6)
• Hypomagnesemia grade 2, N (%)	69 (53)
• Hyponatremia grade 3, N (%)	40 (31)
Relapse	
• Total occurrence of relapse, N (%)	34 (26)
• Median day of onset relapse (range)*	159 (7–1224)
• Relapse at 6 months	19 (15%)
Mortality	
• All-cause mortality	40 (32.5)
• All-cause mortality at 1 year, N (%)	25 (19)
• Non-relapse mortality, N (%)	22 (17)
• Non-relapse mortality at 1 year, N (%)	12 (9)
• Relapse-related mortality, N (%)	20 (15.5)
• Relapse-related mortality at 1 year, N (%)	13 (10)
Overall survival; at 1 year and 3 years	82% and 67%
Relapse-free survival; at 1 year and 3 years	70% and 54%
GvHD/relapse-free survival; at 1 year and 3 years	43% and 31%

*Relative to HCT, where HCT is day 0, and the start of CsA was on day -3.

**Fig 2:**
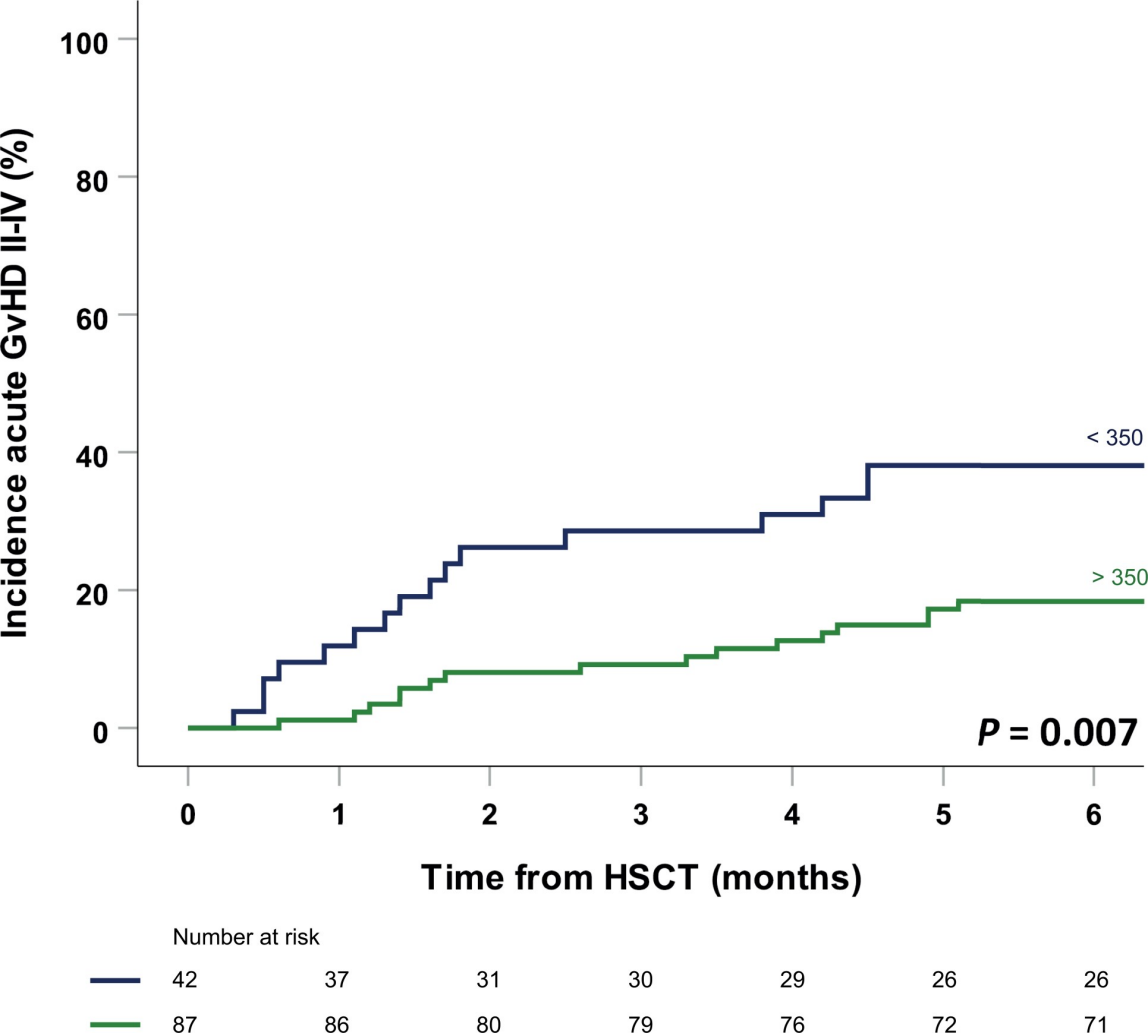
Cumulative prevalence of grade II-IV acute GvHD in patients with a trough CsA concentration <350 ng/mL (blue; N = 42) versus a trough CsA concentration ≥350 ng/mL (green; N = 87).

An analysis of the incidence of aGvHD over time using Kaplan-Meier and Cox regression analysis revealed that both MUD/MMUD and a CsA W1-4 value ≥350 ng/mL had a significant influence in both univariate and multivariate analyses (Table B in S1 File). The incidence of aGvHD within the first 6 months was 18% (16/87) in patients with a CsA W1-4 value ≥350 ng/mL compared to 38% (16/42) in patients with a concentration <350 ng/mL, corresponding to a hazard ratio (HR) of 0.38 (95% CI: 0.19–0.77; *P* = 0.007) (Fig 2). We performed the same analysis for a cut-off value of 300 ng/mL, although there was a significant difference in aGvHD incidence (P = 0.02), the group with levels < 300 ng/ml was too small to derive any conclusions. Nevertheless, this suggests that trough concentrations above 300 ng/mL are acceptable.

Moderate-severe cGvHD occurred in 28% (36/129) of patients. Multivariate logistic regression analysis revealed that a HSCT with a (M)MUD increased and the use of ATG reduced the risk for cGvHD (HR 4.30; 95% CI: 1.45–12.77; *P* = 0.007, and HR 0.33; 0.38; 95% CI: 0.19–0.77; *P* = 0.03)). No difference was seen in the incidence of cGvHD between patients with CsA W1-4 value < or ≥350 ng/mL (33.3% versus 25%; *P* = 0.42).

#### Infection

No impact of CsA trough levels was seen on CMV infection and disease (Table A in S1 File).

#### Acute renal toxicity

Renal toxicity was relatively common among patients in our cohort who received high-dose CsA. Specifically, 49% (63/129) and 7.5% (10/129) of patients developed grade 2 (creatinine 2–3 times above baseline) or grade 3 (>3 times above baseline) renal toxicity (Table 2). Consistent with renal toxicity, creatinine levels began to rise gradually from the day of HSCT, reaching 125% of baseline at a median of 20 days (range: 6–42 days) and peak levels on day 49 (range: 9–117 days). To our surprise, we found no significant association between renal toxicity (i.e., grade ≥2) and CsA W1-4, and no other risk factors reached statistical significance. We also found no significant correlation between CsA W1-4 and the change in creatinine levels relative to baseline (R^2^ = 0.016) (Table A and Figure B in S1 File).

#### Hepatic toxicity

We observed two peaks in hyperbilirubinemia following HSCT. The first peak occurred early at a median of 6 days after HSCT (range: 1–11) and appeared to coincide with the start of CsA treatment. Grade 3 hyperbilirubinemia was present in 29% of patients (38/129) and was often not accompanied by any other abnormal liver function tests. In these 38 patients, the median bilirubin level was 68 μmol/L (range: 51–161 μmol/L), and the hepatic toxicity resolved after the dose of CsA was adjusted. None of the cases of early hyperbilirubinemia were related to either GvHD or sinusoidal obstructive syndrome (SOS). Patient age (i.e., ≥60 years) was the only variable that was significantly associated with an increased risk of hepatic toxicity (HR: 2.39; 95% CI: 1.03–5.54; *P* = 0.04) (Table A in S1 File).

The second peak in hyperbilirubinemia occurred after a median interval of 142 days and was associated with the occurrence of aGvHD with liver involvement in 6% of cases. We found no significant association between this second peak and increased CsA W1-4 concentration.

#### Electrolyte imbalance

Hypomagnesemia (a common side effect) developed in approximately half of our patients; specifically, 53% of patients (69/129) presented with grade 2 hypomagnesemia. In contrast, grade 2 hyperkalemia was relatively rare, occurring in only 6% (8/129) of patients (Table 2). Hypomagnesemia developed relatively early, with a median onset of 7 days (range: 2–19 days). CsA W1-4 concentration was significantly associated with grade 2 hypomagnesemia, occurring in 59% and 34% of patients with a CsA W1-4 value ≥350 ng/mL versus <350 ng/mL, respectively (HR: 2.95; 95% CI: 1. 37–6.33: *P* = 0.05) (Table A in S1 File).

In addition, hyponatremia was also a relatively common and early complication; 31% of patients (40/129) developed grade 3 hyponatremia with a median onset of 11 days (range: 4–41 days) (Table 2). However, we found no association between CsA W1-4—or any other factor—and the occurrence of hyponatremia.

Additional urinalysis had been performed in 19 patients. In 7 of these patients, the apparent cause of hyponatremia was identified and included the use of diuretics and the occurrence of SIADH (syndrome of inappropriate antidiuretic hormone secretion) during an infection; in these cases, the median onset of hyponatremia was relatively late (30 days after HSCT). In the other 12 patients, the median onset of hyponatremia was 9 days after HSCT, and both urine and plasma analyses revealed a pattern consistent with SIADH; specifically, the median plasma sodium level was 125 mmol/L (range: 121–129 mmol/L), the median urine sodium level was 64 mmol/L (range: 44–164 mmol/L), the median plasma osmolality was 267 mOsmol/kg (range: 259–274 mOsmol/kg), and the median urine osmolality was 454 mOsmol/kg (range: 227–654 mOsmol/kg).

#### Post-HSCT outcome

**Relapse.** Among our patient cohort, the rate of relapse was 26% (34/129), occurring after a median delay of 159 days (range: 37–1224 days). Early relapse (defined as occurring within 6 months of HSCT) occurred in 15% of all patients (19/129). Our analysis revealed that both a diagnosis of AML/MDS and a high/very high DRI value were significantly associated with an increased rate of relapse (Table A in S1 File).

**Fig 3:**
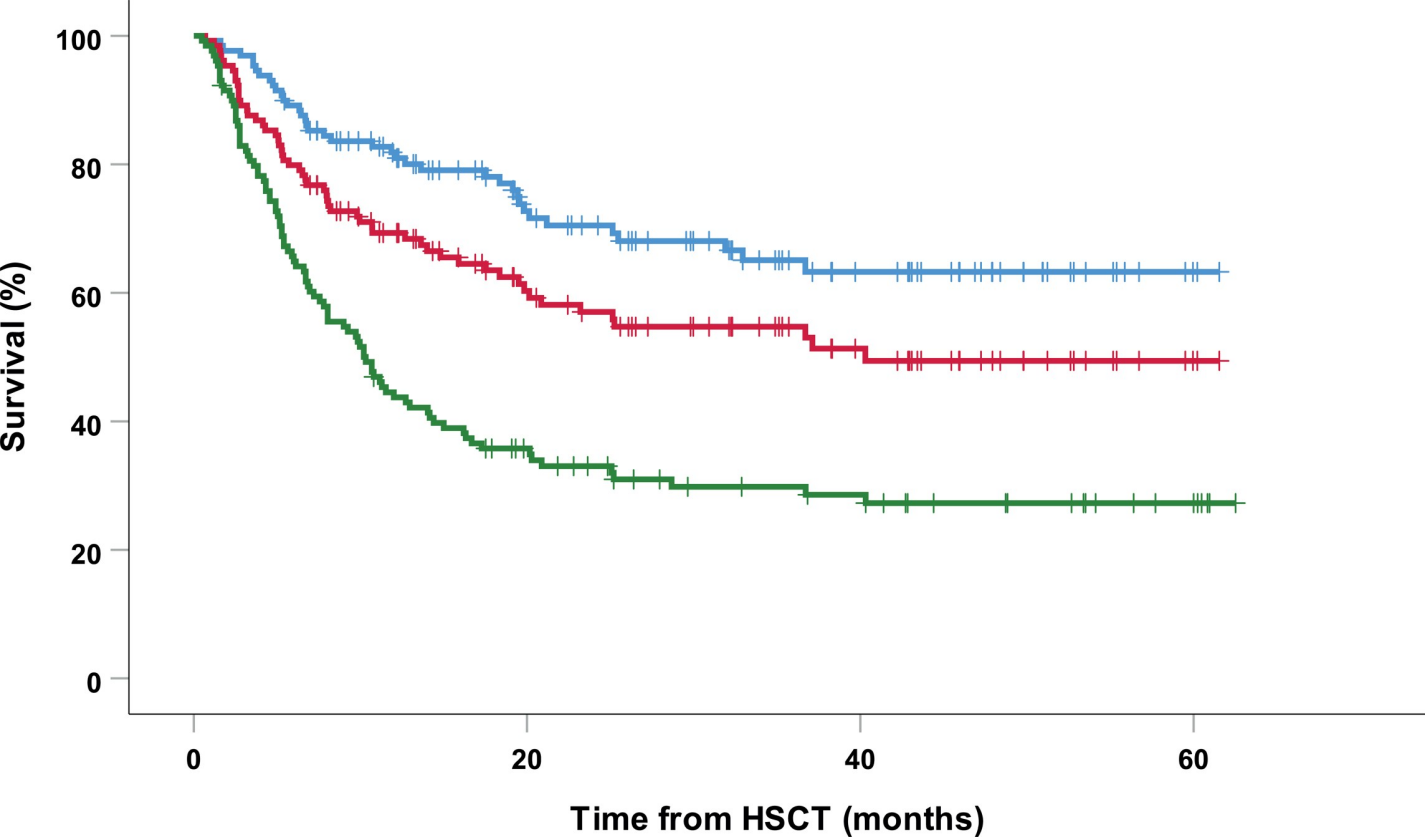
Overall survival, relapse-free, and GvHD/relapse-free survival in the total cohort. Kaplan-Meier curves for OS (blue), RFS (red) and GRFS (green) are shown for the entire cohort of 129 patients who received Flu-TBI‒based non-myeloablative conditioning prior to HCT.

**NRM, RFS, GRFS and OS.** The median follow-up of all 129 patients was 20.6 months (range: 0.7–61.5 months), for patients alive the median follow up was 30.9 months (range: 7.0–61.5). The overall mortality rate was 32.5% (45/129) and occurred after a median post-HSCT interval of 244 days (range: 21–1127 days); relapse-related mortality (RRM) and non-relapse-related mortality (NRM) occurred in 15.5% and 17% of patients, respectively (Table 2). The overall survival (OS) rate at 1 and 3 years was 81% and 69%, respectively, the rate of relapse-free RFS at 1 and 3 years was 70% and 54%, respectively, and the rate of GRFS at 1 and 3 years was 43% and 31% (Table 2 and Fig 3).

No factors were significantly associated with NRM. However, a univariate analysis revealed that both a diagnosis of AML/MDS and a high/very high DRI were associated with reduced RFS and GRFS (Table B in S1 File). On multivariate analysis, only high/very high DRI remained significantly associated with both reduced RFS and GRFS (HR: 2.74 and 2.05, respectively; both *P =* 0.001) (Table B in S1 File). Also with respect to OS, a high/very high DRI was the only significant factor identified by multivariate analysis (HR: 1.98; 95% CI: 1.12–3.68; *P* = 0.02) (Table B in S1 File).

None of the cut-off values for CsA W1-4 were significantly associated with any of the survival outcomes, including NRM, RFS, GRFS and OS (Table B and Figures C and D in S1 File). However, although not statistically significant, the 1-year the GRFS tended to be better in patients with CsA W1-4 ≥350 ng/mL versus <350 ng/mL (47% vs 38%).

## Discussion

At our medical center, we historically used a target trough concentration of CsA of 200–400 ng/mL for allogeneic HSCT recipients following NMA conditioning, in accordance with established guidelines[9]. Although this target is generally recommended, there is a striking paucity of data supporting this target range; moreover, several studies showed a clear reduction in the incidence of aGvHD among patients who have a higher trough concentration in the first few weeks following HSCT[12–15]. In our study, more than 95% of patients had a CsA trough concentration >200 ng/mL, with a median CsA W1-4 value of 402.5 ng/mL. This suggests that with respect to aGvHD prophylaxis, so-called “optimal” CsA dosing was achieved in virtually all patients, at least based on current recommendations. Nevertheless, we found that achieving a CsA W1-4 concentration ≥350 ng/mL significantly reduced the occurrence of grade II-IV aGvHD within the first 6 months following HSCT. This protective effect was the most prominent early after HSCT, as illustrated by the strong divergence in the occurrence of aGvHD and OS after the first two months (see Figs 2 and 3). Similar results were reported by Malard *et al*.[12]; their optimal CsA trough concentration was 348 ng/mL. Although this difference in optimal concentration may be due in part to differences in the clinical settings and/or the cohort composition, it suggests that a target CsA trough concentration of just ≥200 ng/mL is too low for aGvHD prophylaxis. Our findings are also supported by a study by Rogosheske *et al*., who showed that each 50 ng/mL increase in trough CsA concentration was associated with a 33% decrease in the relative risk of developing aGvHD[15]. In concordance with the aforementioned and previous studies, we found no effect of CsA levels on cGVHD[15, 32].

No significant correlation between CsA W1-4 concentration and any of the survival outcomes measured was found. In an early study by Bacigalupo, a higher risk of relapse and reduced disease-free survival was seen in patients who received a myeloablative conditioning regimen and had higher median CsA levels in the first 2 weeks after transplant[33]. Surprisingly, RFS did not differ between CsA W1-4 groups in our population. However, only a fraction of our patients had CsA levels > 600ng/ml which was the median CsA level associated with higher risk of relapse in that study. Despite having an apparently high impact on the risk of aGvHD, in our cohort, NRM was also not influenced. This finding is in contrast with a study by Ram *et al*., which found decreased risks of aGVHD NRM and overall mortality with a higher CsA trough concentration in the first 2 weeks after transplantation. [17]. The absence of such an effect in our study is likely related to the overall low rate of NRM (9% at 1 year) and/or the relatively low rate of grade III-IV aGvHD (10%) in our cohort. Importantly, our study concerned patients transplanted in more recent years (between 2013–2017 versus 2001–2009), and changes in supportive care and aGvHD treatment might at least partially explain this difference.

In our population, we found no clear correlation between CsA trough concentration and the occurrence of either hepatic or renal toxicity. Although the incidence of hepatic and renal toxicity was relatively high, it is similar to previous results reported for patients who received Flu-TBI‒based conditioning[4, 18]. In addition, although CsA-related renal and hepatic toxicity was shown to be dose-dependent in previous studies, particularly in kidney transplant recipients[34, 35], we found no such effect in our cohort. One possible explanation might be the relatively high starting dose of CsA (4.5 mg/kg twice daily) in our cohort; at these high dosages, individual factors such as previous renal toxicity, concomitant drug use, and age likely become more important factors in determining a patient’s susceptibility to develop toxicity. For example, our finding that nearly 50% of patients in our cohort developed grade 2 renal toxicity may have been due—at least in part—to the relatively high median age of this cohort (61 years), given that the risk of nephrotoxicity increases with age[36].

Hyperbilirubinemia was an isolated finding that occurred early following HSCT and resolved after the CsA dose was adjusted; none of these early cases were attributed to liver GvHD or SOS. *In vitro* studies offer a possible explanation for this effect, showing that CsA inhibits canalicular bile transport by inhibiting the bile salt export pump, resulting in cholestasis and decreased bilirubin excretion[4, 37]. Moreover, the decrease in renal function may have contributed to hyperbilirubinemia by reducing renal clearance of conjugated bilirubin.

A higher CsA concentration (i.e., ≥350 ng/mL) was associated with the occurrence of hypomagnesemia. Although we found a statistically significant difference in magnesium concentration between patients with a CsA concentration >350 ng/mL compared to patients with a CsA concentration <350 ng/mL, the overall rate of hypomagnesemia in our cohort was relatively high. Thus, decreased magnesium reabsorption—with loss of renal magnesium due to tubulopathy—is highly common among patients treated with CsA and appears to be concentration-dependent[38]. Because of the limited clinical consequences associated with this side effect, we believe that adjusting the dose of CsA based solely on the development of hypomagnesemia may not be necessary.

A relatively high percentage of our patients (31%) developed grade 3 hyponatremia; this finding is somewhat remarkable given that hyponatremia is usually considered to be related to the use of TAC rather than CsA[39]. The mechanism by which calcineurin inhibitors induce hyponatremia is poorly understood and appears to differ between CsA and TAC. For example, TAC has been shown to increase renal sodium excretion and SIADH, both of which contribute to hyponatremia in kidney transplant and allogeneic HSCT recipients[32, 39–41]. With respect to CsA, although evidence argues against renal salt wasting, virtually no data exists regarding the role of SIADH[41]. In our cohort, CsA was implicated as the cause of hyponatremia, with urinalysis suggesting SIADH as the underlying mechanism of action. However, the lack of urinalysis results for half of the patients with hyponatremia precludes any conclusions regarding the possible relationship between hyponatremia and CsA. Additional studies are needed in order to examine this putative association further and to test the hypothesis that SIADH is the underlying mechanism of action.

Our study was limited by its retrospective nature and by the fact that it included patients from a single medical center. Differences in the composition of patient cohorts among various centers and/or studies can play a role in this type of analysis, thus preventing us from making any general recommendations regarding CsA target values. Still, our finding that the rate of aGvHD was lower among patients with a higher trough CsA concentration suggests that the existing target range may be too low and that the optimal target CsA concentration should be set to ≥350 ng/mL for the first month after transplantation. Importantly, in our relatively older cohort of patients, this higher concentration was not associated with a clinically relevant increased risk of toxicity or an increase in risk for relapse. At the very least, adjusting the CsA merely based on exceeding the current upper limit of 400 ng/mL should be discouraged. In addition, dose augmentation should be considered in patients with an early trough concentration below 300–350 ng/mL, particularly if there is no evidence of toxicity. Nevertheless, prospective data are needed in order to support our findings and recommendations.

In conclusion, we report a significant association between post-HSCT trough concentration of CsA and the occurrence of aGvHD, and we report that trough CsA concentration is not generally associated with common forms of post-HSCT toxicity. Our findings, supported by previous studies suggest that 350–500 ng/mL is a more appropriate target concentration for CsA trough levels in the first few weeks following HSCT, representing a paradigm shift from the current recommendation of 200–400 ng/mL. This change in the target CsA trough concentration is particularly relevant in the context of Flu-TBI‒based NMA conditioning in which GvHD prophylaxis includes an initial dosing regimen of oral CsA given at 4.5 mg/kg twice daily.

## Supporting information

S1 File S1 supplementary file. (DOCX)
