# Supplementary material for: Cyclosporine A trough concentrations are associated with acute GvHD after non-myeloablative allogeneic hematopoietic cell transplantation
Source: PLoS One. 2019 Mar 21;14(3):e0213913. doi: 10.1371/journal.pone.0213913 (PMC6428294; doi:10.1371/journal.pone.0213913)
Supplement: S1 File — (DOCX) [file pone.0213913.s001.docx]

**Supplementary file S1**

**Patient and methods:**

**Supportive care measures**

Patients who received decitabine and/or ATG received antimicrobial prophylaxis consisting of 500 mg ciprofloxacin twice daily. Patients who had a positive serology test for herpes simplex virus and/or varicella zoster virus received 500 mg valacyclovir twice daily. Antifungal prophylaxis was not routinely given during the conditioning treatment or neutropenic phase. After repopulation, all patients received *Pneumocystis jirovecii* prophylaxis consisting of 480 mg/day co-trimoxazole. Weekly monitoring for CMV and EBV reactivation was performed using PCR-based screening of blood plasma.


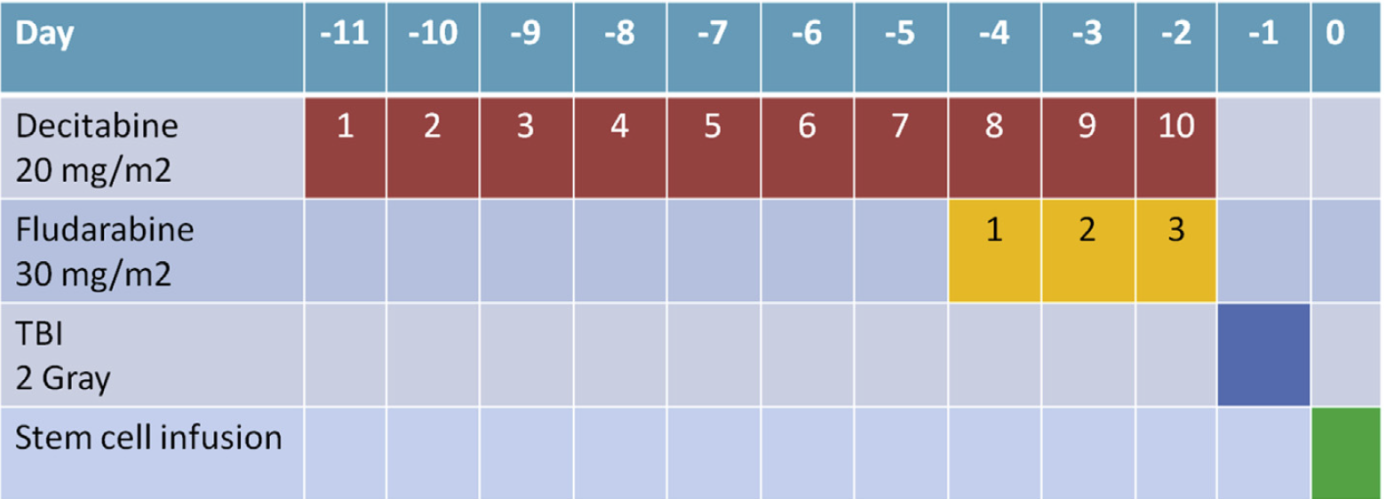


**Supplementary Figure A:** Flu-TBI‒based conditioning regimen. For all patients, flurabadine and TBI were administered on the indicated days, and HCT was performed on day 0; 43 patients received decitabine on the indicated days. In mismatched unrelated transplants, ATG 2 mg/kg/day was also administered on days -8, -7, -6, and -5^1^.


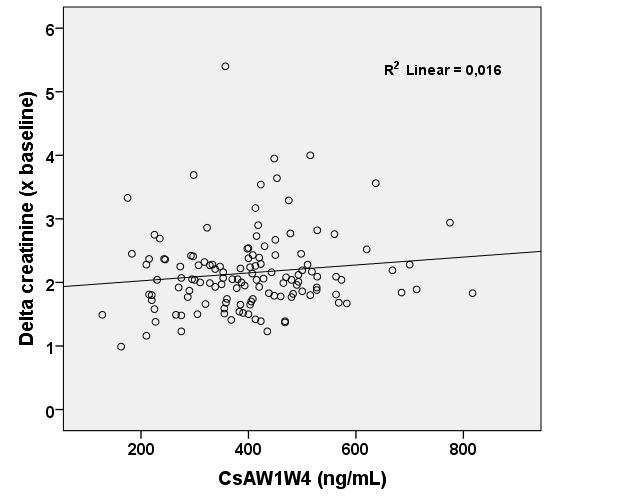


**Supplementary Figure B:** CsA W1-4 was not correlated with the change in creatinine levels relative to baseline.


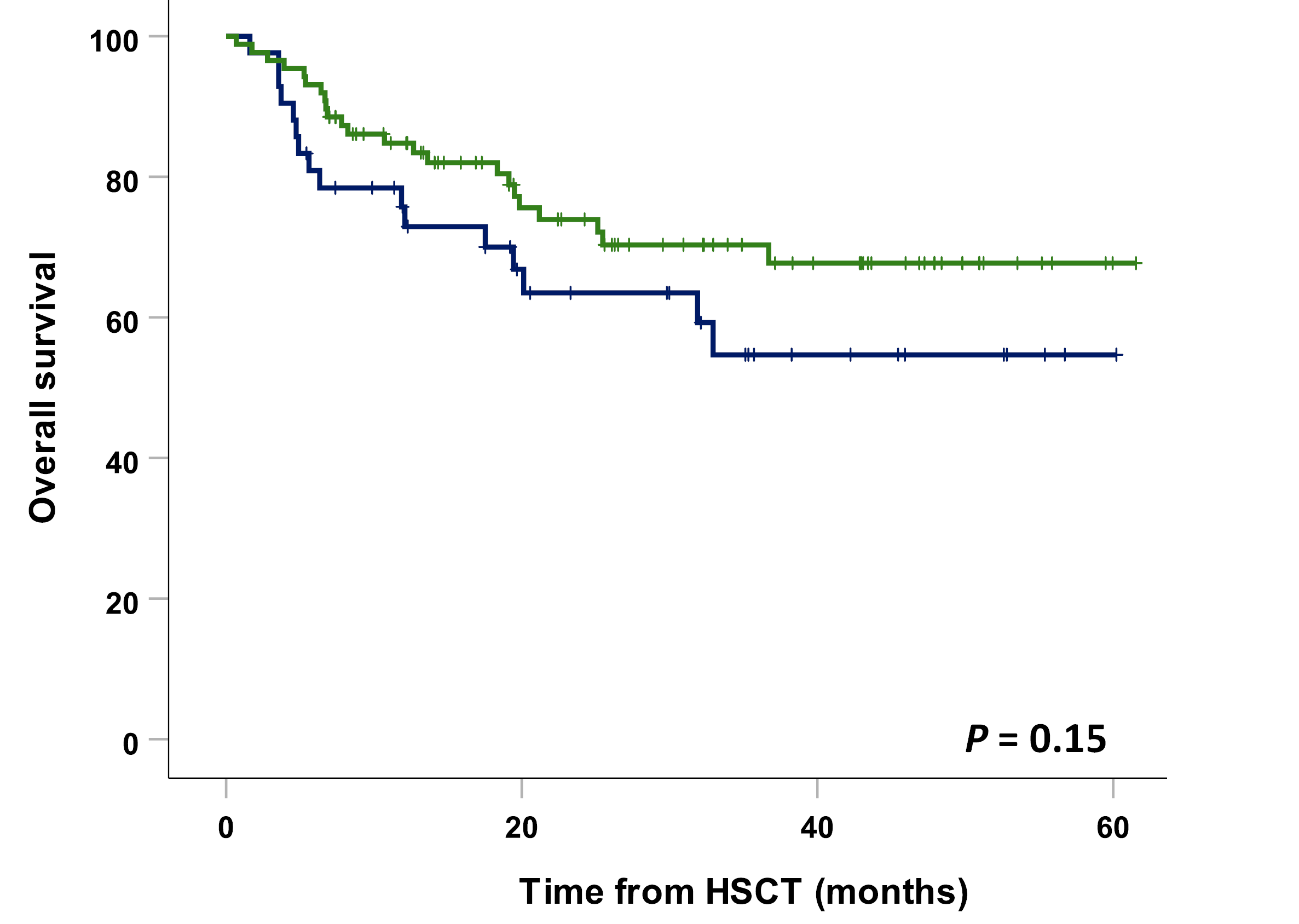


**Supplementary Figure C:** Overall survival (OS) in patients with a trough CsA concentration <350 ng/mL (blue; N=42) versus a trough CsA concentration ≥350 ng/mL (green; N=87).


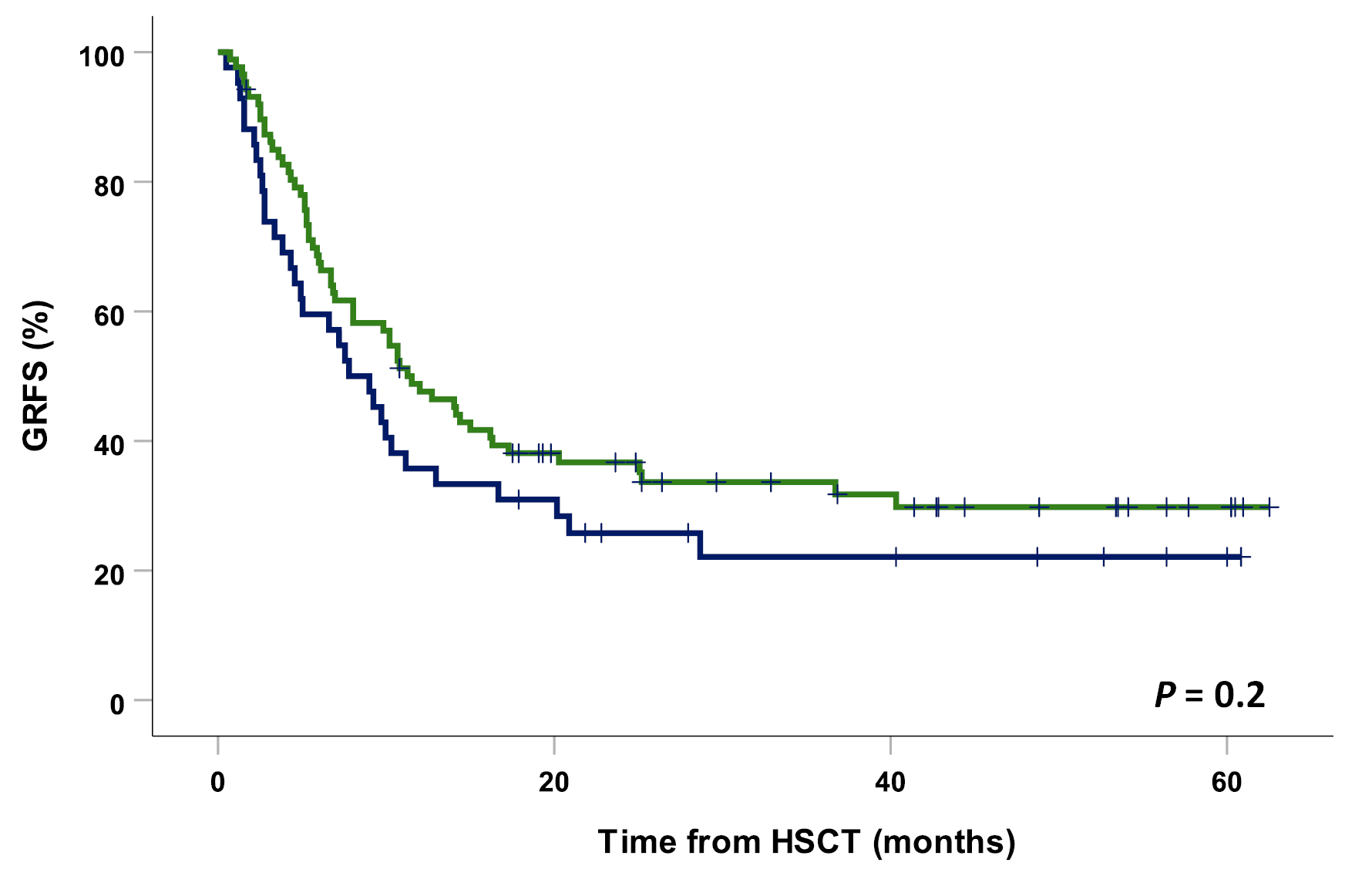


**Supplementary Figure D:** GvHD/relapse-free survival (GRFS) in patients with a trough CsA concentration <350 ng/mL (blue; N=42) versus a trough CsA concentration ≥350 ng/mL (green; N=87).

**Supplementary Table A:** Univariate and multivariate analyses of risk factors for post-HCT complications.

| **Outcome variable** | **Univariate** | | | **Multivariate** | | |
| --- | --- | --- | --- | --- | --- | --- |
| **GvHD II-IV on day 180** | **HR** | **95% CI** | ***P*-value** | **HR** | **95% CI** | ***P*-value** |
| Conditioning + ATG | - | - | - | - | - | - |
| Age >60 years | - | - | - | - | - | - |
| Patient CMV-pos status | - | - | - | - | - | - |
| Donor (M)MUD vs MRD | 3.3 | 1.06-10.19 | 0.04 | 3.57 | 1.12-11.30 | 0.03 |
| CsA ≥350 ng/mL | 0.37 | 0.16-0.84 | 0.02 | 0.34 | 0.15-0.80 | 0.01 |
| Diagnosis AML-MDS vs other | - | - | - | - | - | - |
| Gender F vs M | - | - | - | - | - | - |
| **Moderate + severe cGvHD** |  |  |  |  |  |  |
| Conditionering + ATG | 0.38 | 0.10-1.38 | NS 0.14 | 0.33 | 0.61-0.89 | 0.03 |
| Age >60 years | 0.54 | 0.25-1.18 | NS 0.12 | - | - | - |
| Donor (M)MUD vs MRD | 2.95 | 1.04-8.35 | 0.04 | 4.30 | 1.45-12.77 | 0.08 |
| CsA ≥350 ng/ml | - | - | - | - | - | - |
| CsA ≥500 ng/ml | - | - | - | - | - | - |
| Diagnosis AML-MDS vs other | - | - | - | - | - | - |
| Gender F vs M | - | - | - | - | - | - |
| **Hypomagnesemia <0.5 mmol/L** |  |  |  |  |  |  |
| Conditionering + ATG | - | - | - | - | - | - |
| Age >60 years | - | - | - | - | - | - |
| Donor (M)MUD vs MRD | - | - | - | - | - | - |
| CsA ≥350 ng/ml | 2.95 | 1.37-6.33 | 0.05 | - | - | - |
| CsA ≥500 ng/ml | 2.67 | 1.03-6.94 | 0.04 | - | - | - |
| Diagnosis AML-MDS vs other | 0.48 | 0.20-1.13 | NS 0.13 | - | - | - |
| Gender F vs M | - | - | - | - | - | - |
| **Hyponatremia <130 mmol/L** |  |  |  |  |  |  |
| Conditioning + ATG | - | - | - | - | - | - |
| Age >60 years | - | - | - | - | - | - |
| Donor (M)MUD vs MRD | - | - | - | - | - | - |
| CsA ≥350 ng/ml | - | - | - | - | - | - |
| CsA ≥500 ng/ml | - | - | - | - | - | - |
| Diagnosis AML-MDS vs other | - | - | - | - | - | - |
| Gender F vs M | - | - | - | - | - | - |
| **Hyperbilirubenemia** |  |  |  |  |  |  |
| Conditioning + ATG | - | - | - | - | - | - |
| Age >60 years | 2.62 | 1.14-6.02 | 0.03 | 2.39 | 1.03-5.54 | 0.04 |
| Donor (M)MUD vs MDS | - | - | - | - | - | - |
| CsA ≥350 ng/ml | - | - | - | - | - | - |
| CsA ≥500 ng/ml | - | - | - | - | - | - |
| Diagnosis AML-MDS vs other | 0.42 | 0.15-1.22 | NS 0.08 | - | - | - |
| Gender F vs M | - | - | - | - | - | - |
| **Creatinine change (≥times 2)** |  |  |  |  |  |  |
| Conditioning + ATG | - | - | - | - | - | - |
| Age >60 years | - | - | - | - | - | - |
| Donor (M)MUD vs MRD | - | - | - | - | - | - |
| CsA ≥350 ng/ml | - | - | - | - | - | - |
| CsA ≥500 ng/ml | - | - | - | - | - | - |
| Diagnosis AML-MDS vs other | - | - | - | - | - | - |
| Gender F vs M | - | - | - | - | - | - |
| **CMV infection** |  |  |  |  |  |  |
| Conditioning + ATG | - | - | - | - | - | - |
| Age >60 years | - | - | - | - | - | - |
| Patient CMV-pos status | 7.72 | 2.16-27.56 | 0.001 | - | - | - |
| Donor (M)MUD vs MRD | - | - | - | - | - | - |
| CsA ≥350 ng/ml | - | - | - | - | - | - |
| CsA ≥500 ng/ml | - | - | - | - | - | - |
| Diagnosis AML-MDS vs other | - | - | - | - | - | - |
| Acute GvHD day 180 | - | - | - | - | - | - |
| **Relapse 6 months** |  |  |  |  |  |  |
| Conditioning + ATG | - | - | - | - | - | - |
| Age >60 years | - | - | - | - | - | - |
| Patient CMV-pos status | - | - | - | - | - | - |
| Donor (M)MUD vs MRD | 0.49 | 0.21-1.13 | NS 0.12 | - | - | - |
| CsA ≥350 ng/ml | - | - | - | - | - | - |
| CsA ≥500 ng/ml | - | - | - | - | - | - |
| Diagnosis AML-MDS vs other | 3.30 | 1.36-8.00 | 0.014 | 4.42 | 1.65-11.88 | 0.003 |
| Gender F vs M | - | - | - | - | - | - |
| DRI high/very high | 4.46 | 1.94-10.26 | <0.001 | 4.77 | 1.92-11.86 | 0.001 |

**Supplementary Table B:** Univariate and multivariate analyses of risk factors for post-HCT outcome

| **Outcome variable** | **Univariate** | | | **Multivariate** | | |
| --- | --- | --- | --- | --- | --- | --- |
| **GvHD** | **HR** | **95% CI** | ***P*-value** | **HR** | **95% CI** | ***P*-value** |
| Conditioning + ATG | - | - | - | - | - | - |
| Age >60 years | - | - | - | - | - | - |
| Patient CMV-pos status | - | - | - | - | - | - |
| Donor (M)MUD vs MRD | 2.90 | 1.02-8.26 | 0.047 | 3.09 | 1.08-8.81 | 0.035 |
| CsA ≥350 ng/mL | 0.40 | 0.20-0.841 | 0.01 | 0.39 | 0.19-0.77 | 0.007 |
| Diagnosis AML-MDS vs other | - | - | - | - | - | - |
| Gender F vs M | - | - | - | - | - | - |
| DRI high/very high | - | - | - | - | - | - |
| **NRM** |  |  |  |  |  |  |
| Conditioning + ATG | - | - | - | - | - | - |
| Age >60 years | - | - | - | - | - | - |
| Patient CMV-pos status | - | - | - | - | - | - |
| Donor (M)MUD vs MRD | - | - | - | - | - | - |
| CsA ≥300 ng/ml | - | - | - | - | - | - |
| Diagnosis AML-MDS vs other | - | - | - | - | - | - |
| Acute GvHD day 180 | - | - | - | - | - | - |
| Gender F vs M | 2.817 | 0.86-7.25 | NS 0.09 | - | - | - |
| DRI high/very high | - | - | - | - | - | - |
| HCT-CI ≥3 | - | - | - | - | - | - |
| **RFS** |  |  |  |  |  |  |
| Conditioning + ATG | - | - | - | - | - | - |
| Age >60 | - | - | - | - | - | - |
| Patient CMV-pos status | - | - | - | - | - | - |
| Donor (M)MUD vs MRD | 0.69 | 0.40-1.21 | NS 0.2 | - | - | - |
| CsA ≥350 ng/ml | - | - | - | - | - | - |
| CsA ≥500 ng/ml | 1.6 | 0.87-2.83 | NS 0.14 | - | - | - |
| Diagnosis AML-MDS vs other | 1.64 | 0.93-2.90 | NS 0.09 | 1.76 | 0.97-3.17 | NS 0.06 |
| Gender F vs M | - | - | - | - | - | - |
| DRI high/very high | 2.90 | 1.70-4.96 | <0.001 | 2.74 | 1.57-4.77 | <0.001 |
| **GRFS** |  |  |  |  |  |  |
| Conditioning + ATG | 0.54 | 0.29-1.02 | NS 0.06 | 0.55 | 0.29-1.04 | NS 0.06 |
| Age >60 | - | - | - | - | - | - |
| Patient CMV-pos status | - | - | - | - | - | - |
| Donor (M)MUD vs MRD | - | - | - | - | - | - |
| CsA ≥350 ng/ml | - | - | - | - | - | - |
| CsA ≥500 ng/ml | - | - | - | - | - | - |
| Diagnosis AML-MDS vs other | 1.44 | 0.90-2.31 | NS 0.13 | 1.62 | 0.99-2.61 | NS 0.05 |
| Gender F vs M | - | - | - | - | - | - |
| DRI high/very high | 2.00 | 1.29-3.06 | 0.002 | 2.05 | 1.33-3.18 | 0.001 |
| **OS** |  |  |  |  |  |  |
| Conditioning + ATG | - | - | - | - | - | - |
| Age >60 years | - | - | - | - | - | - |
| Patient CMV-pos status | - | - | - | - | - | - |
| Donor (M)MUD vs MRD | - | - | - | - | - | - |
| CsA ≥350 ng/ml | 0.63 | 0.33-1.19 | NS 0.15 | - | - | - |
| Diagnosis AML-MDS vs other | - | - | - | - | - | - |
| Acute GvHD day 180 | 1.55 | 0.78-3.06 | NS 0.2 | - | - | - |
| Gender F vs M | - | - | - | - | - | - |
| DRI high/very high | 2.05 | 1.06-3.95 | 0.03 | 1.98 | 1.12-3.68 | 0.02 |
| HCT-CI ≥3 | - | - | - | - | - | - |

1. Cruijsen M, Hobo W, van der Velden WJ, Bremmers ME, Woestenenk R, Bar B *et al.* Addition of 10-Day Decitabine to Fludarabine/Total Body Irradiation Conditioning is Feasible and Induces Tumor-Associated Antigen-Specific T Cell Responses. *Biology of blood and marrow transplantation : journal of the American Society for Blood and Marrow Transplantation* 2016; **22**(6)**:** 1000-1008. doi: 10.1016/j.bbmt.2016.02.003
